# Closure of oroantral fistula using platelet rich fibrin with endoscopic middle meatal antrostomy

**DOI:** 10.1186/s12903-024-04409-0

**Published:** 2024-06-16

**Authors:** Amira Tarek Elgabarty, Ahmed Salah Elmahallawy, Ahmed Aly Ibraheam, Mona Samy Oraby

**Affiliations:** 1 Oral and Maxillofacial Surgery Department, Faculty of Dentistry, Alexandria University, Champlion St, Azrite, Alexandria, Egypt; 2 Otorhinolaryngology and skull base surgery Department, Faculty of Medicine, Alexandria University, Champlion St, Azrite, Alexandria, Egypt

**Keywords:** PRF membrane, Oroantral fistula, Endoscopic middle meatal antrostomy

## Abstract

**Background:**

Oroantral fistula (OAF) involves pathological, epithelialized, and unnatural communication between the maxillary sinus and oral cavity. Recently, functional endoscopic sinus surgery has provided minimally invasive treatment options with fewer postoperative complications. The aim of the study was to evaluate the one-stage endoscopic middle meatal antrostomy (EMMA) technique with the application of a platelet-rich fibrin membrane (PRF) for OAF closure and maxillary sinusitis relief.

**Patients and methods:**

Patients who suffered from OAF with odontogenic sinusitis were included in this study. Complete excision of the epithelial tract and any necrotic tissue was performed with proper curettage. Then, EMMA was performed with simultaneous closure of the OAF by the application of PRF membranes that were fixed by sutures and covered with an acrylic splint. Patients were clinically evaluated for OAF closure, pain level, and symptom relief. Additionally, the size of the bone defect was measured with the aid of computed tomography (CT) preoperatively and after 24 weeks postoperatively.

**Results:**

This study included nine eligible patients with a mean age of 38 years. The data were collected, tabulated, and statistically analyzed. Soft tissue healing and bone formation occurred in all patients who achieved maxillary sinusitis relief without any complications. Additionally, pain was significantly lower on the 7th postoperative day than on the 1st postoperative day, according to the statistical analysis of the results (*p* < .001).

**Conclusions:**

One-stage EMMA with the application of PRF membranes and acrylic splint represents a reliable alternative technique for OAF closure and maxillary sinusitis relief that is associated with a lower incidence of complications and minimal postoperative pain.

**Trial registration:**

The trial was registered on 28/02/2024, at clinicaltrials.gov (ID: NCT06281873).

## Introduction

Oroantral fistula (OAF) is a pathological epithelialized communication between the maxillary antrum and the oral cavity. Symptoms of OAF include fluid regurgitation, pain, swelling, and chronic maxillary sinus (MS) diseases [1]. The most common cause of OAF is a complication of maxillary posterior tooth extraction due to the proximity of the roots to the MS in some cases. There are other causes, such as periapical infection from the posterior teeth, maxillary cysts, implants displaced into the MS, tumors, osteoradionecrosis, and trauma. If the OAF is left untreated, microbial flora will pass into the MS, causing infections and sinusitis [1, 2]. 

Any size and duration of the fistula could result in MS infection and sinusitis, which has been reported in the literature [3]. For many years, the Caldwell-Luc operation has been a standard intraoral surgical approach to the cavity of the MS for removal of any foreign body (tooth or root), OAF, dental cyst, maxillary sinusitis, and complete removal of the damaged mucosal lining of the MS [4]. Usually, an antrostomy at the inferior meatus is performed for drainage. This technique has many complications, such as bleeding, postoperative pain, edema, epistaxis, numbness of the face, and sometimes adjacent tooth damage while a hole is made into the sinus cavity [5]. 

Advanced functional endoscopic sinus surgery (FESS) is a minimally invasive technique that has almost completely replaced the radical Caldwell-Luc technique [6]. Currently, endoscopic middle meatal antrostomy (EMMA) is one of the most commonly performed endoscopic sinus surgery procedures for the treatment of chronic and recurrent acute sinusitis, based on the concept that most diseases of the paranasal sinuses are caused by obstruction of the middle meatus region. This technique allows perfect visualization of the MS, and enables reestablishment of the patency of the natural MS ostium and osteomeatal complex with fewer postoperative complications [6, 7]. 

Different surgical and nonsurgical techniques have been developed for the management of OAF. There are various nonsurgical techniques for OAF closure, such as N-butyl cyanoacrylate gel, acrylic splints, laser light, PRF, and metal plates have been proposed in the literature [8, 9]. Surgical interventions such as soft tissue flaps, include buccal advancement flaps (BAFs), palatal pedicle flaps, and distant flaps. The BAF was first introduced by Rehrmann in 1936. It is the most popular technique due to its simplicity and reliability. Additionally, auricular cartilage or bone grafts (e.g., from the retromolar region or chin) have been used for the closure of the OAF. The appropriate technique was selected based on the case and defect size [9, 10]. There are considerable disadvantages associated with these surgical procedures, such as the risk of donor site morbidity, a reduction in vestibular sulcus depth, and discomfort among patients [11]. Recently, the conservative flapless surgical technique was proposed to minimize edema, and postoperative pain. The flapless techniques for OAF closure already documented in the literature are based on the application of a material such as platelet-rich fibrin (PRF) to stabilize the blood clot [12, 13]. 

PRF is regarded as a second generation of platelet concentrate. It is an autologous biomaterial that creates a strong fibrin matrix. It contains concentrations of platelets, growth factors, circulating stem cells, and leukocytes [14, 15]. In recent years, PRF has attracted attention in different medical societies because it accelerates the healing of bones and soft tissue [16, 17]. In the oral and maxillofacial regions, PRF is frequently used for instance, in soft tissue surgery, OAF closure, sinus perforation, socket preservation, and treatment of osteonecrosis of the jaws [15, 18]. 

Few clinical studies on this combined treatment technique have been published. Therefore, additional studies are highly recommended to assess its success rate. We hypothesized that the use of one-stage EMMA combined with the application of PRF membranes and acrylic splint would be effective for OAF closure and maxillary sinusitis relief. The hypothesis of this technique is that controlling the infection and excessive secretions in the affected MS are mandatory for long-term successful closure of the OAF, and sinusitis relief, which is achieved through a minimally invasive EMMA technique to maintain the patency of the MS ostium and sinus drainage with closure of the OAF by the application of autogenous material such as PRF membranes to minimize surgical trauma, and postoperative pain, and to obtain the benefits of PRF, which contains growth factors that stimulate the wound healing process and bone formation.

The aim of this clinical and radiographic study was to evaluate the one-stage EMMA technique in conjunction with the application of PRF membranes and an acrylic splint for OAF closure and maxillary sinusitis relief.

## Patients and methods

### Sample size and study design

The sample size was estimated assuming 5% alpha error and 80% study power. The minimum sample size was calculated to be 8 patients, which was increased to 9 patients to compensate for patients lost to follow-up [13]. The sample size was based on Rosner’s method [20] and calculated by G*Power 3.1.9.7 software [21]. Nine patients of both genders were selected from the outpatient clinic of Alexandria University Teaching Hospital, Egypt. Patients enrolled in this study between February 2023 and February 2024 were selected after they fulfilled the inclusion criteria.

### The study’s inclusion criteria were as follows

1. Adult patients without a preference for a certain sex.
2. Patients who suffered from chronic OAF that occurred after the extraction of upper posterior teeth with radiographic evidence of sinus involvement.

### The exclusion criteria

1. Patients who had any systemic disease that contraindicated any intervention under general anesthesia (e.g., coronary artery disease) or disease that impaired the healing process (e.g., uncontrolled diabetes mellitus).
2. Patients with an OAF that occurred as a result of other causes (e.g., cysts, trauma, or tumors).

### Ethical approval and informed consent

In this study, all procedures were approved by the Institutional Review Board (IRB) of the Research Ethics Committee at the Faculty of Dentistry, Alexandria University, Egypt (IRB No.001056 – IORG 0008839) after confirming that all the procedures followed the Helsinki Declaration [19]. The trial has been registered on clinicaltrials.gov (ID: NCT06281873). Before the operation, each patient received information about the operation and probable postoperative complications. All patients signed a well-informed consent form.

## Methods

### Preoperative assessment and clinical examination

#### History

A detailed patient history was obtained including name, age, sex, residence, past medical, and dental history. Additionally, the chief complaint was documented including the time, date, and presence of pain or headache.

#### Clinical examination

Extraoral and intraoral examinations were performed for each patient by inspection and palpation.

##### **Extraoral examination**

- Fluid regurgitation from the nose.
- Nasal obstruction or discharge from the ipsilateral nostril.

##### **Intraoral examination**

- Determine the causative tooth and the location of the OAF.
- Pus discharge from the fistula.
- The Valsalva maneuver was performed by asking the patient to hold his nose closed, to close his mouth, and to try to blow gently. Then, he was asked to open his mouth immediately to check for any bubbles that had formed at the suspected location of the OAF [11].

#### Radiographic examination

For proper assessment and treatment planning, a computed tomography (CT) scan was performed preoperatively for all patients to detect any abnormalities or opacity in the paranasal sinuses, in addition to any roots or foreign bodies that may be displaced into the sinus.

### The surgical procedure

#### Preoperative patient preparation

In long-standing OAF associated with purulent discharge, the sinus was irrigated daily with povidone iodine (Betadine: manufactured by the Nile Company, Egypt) mouthwash through the fistula, and a broad-spectrum antibiotic was prescribed for 5 days according to culture and sensitivity tests. All the tests were sensitive to amoxycillin and clavulanic acid (Augmentin 1 g: 875 mg of amoxycillin and 125 mg of clavulanic acid, manufactured by GlaxoSmithKline, UK).

#### Acrylic splint fabrication

An alginate impression was taken preoperatively for the upper arch, and it was poured with dental stone. Then, a well-fitted acrylic splint was constructed on the stone model. The fitting surface of the acrylic splint was relieved, highly polished, and immersed in povidone iodine solution prior to insertion.

#### Operative procedure

**Fig. 1:**
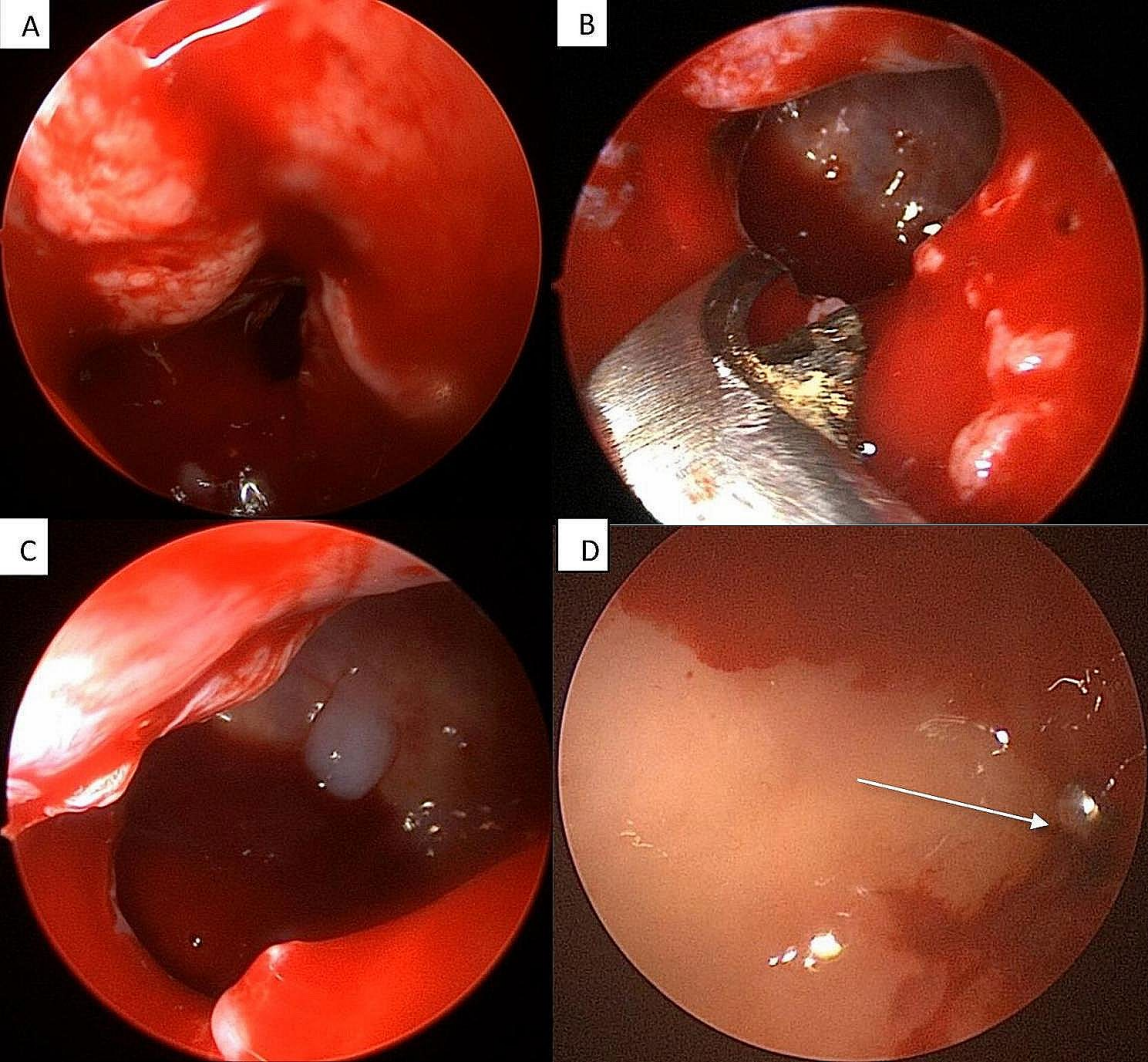
Clinical photos show: **(A)** Endonasal endoscopy image showing a blocked ostium. **(B)** Endoscopic image showing the widened natural maxillary ostium. **(C)** The inflamed MS mucosa with excessive secretions. **(D)** Examination of the MS cavity, and the site of the OAF from above with visualization of a penetrated blunt instrument inserted from the oral cavity

All patients underwent surgery under general anesthesia. The surgeon made an intraoral incision around the orifice of the OAF (fistulectomy). The epithelial tract, unhealthy bone, and any necrotic tissue within the opening of the fistulous tract were removed completely and excised with proper curettage. For MS surgery, a 4.0 mm diameter rigid endonasal endoscope (Karl Storz)^1^ was used. Then, an uncinectomy was performed, followed by a middle meatal antrostomy. The MS cavity was properly examined through the enlarged natural MS ostium, and all the excessive mucus secretions was drained. However, there was no need for meticulous stripping of the mucosa as much as possible; only polyps were removed if observed. Then, the MS cavity was copiously irrigated with saline (Fig. 1).

## Protocol of PRF membrane preparation

**Fig. 2:**
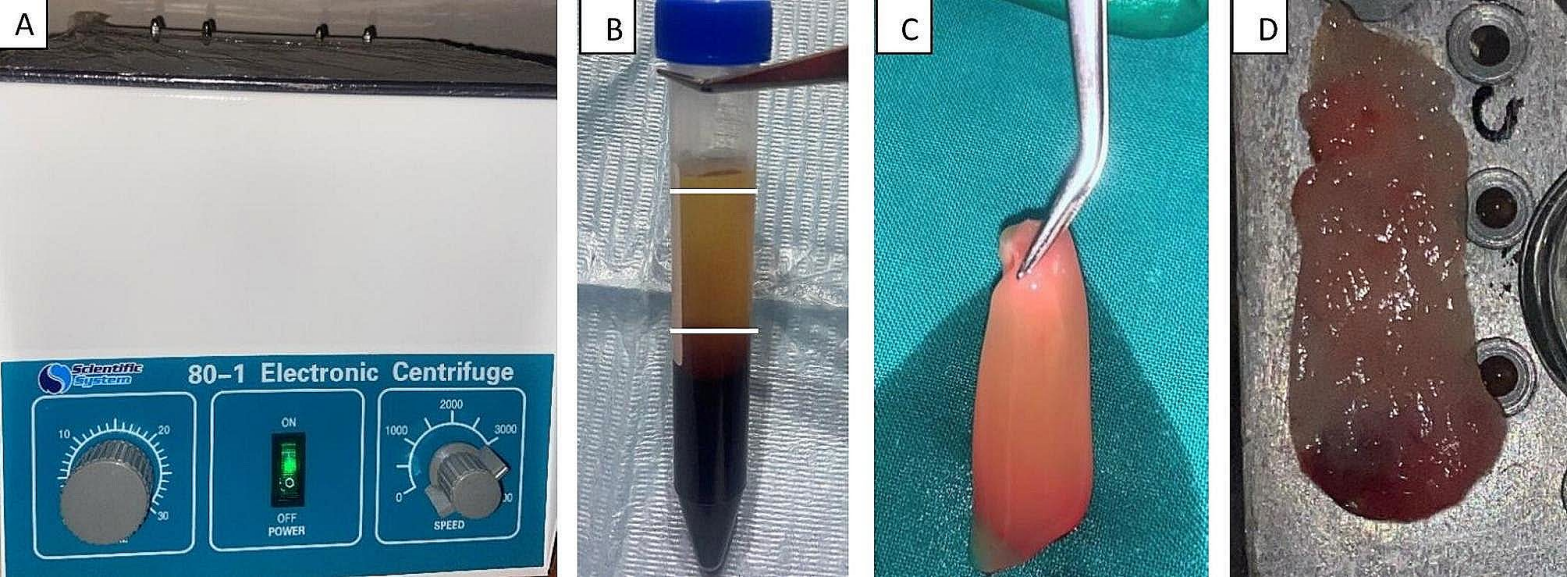
The pictures show the stages for the preparation of the PRF membrane: **(A)** Centrifuge device. **(B)** Production of a PRF clot in a blood collection tube after centrifugation: PRF in the middle of the tube, RBCs collected at the base, and acellular plasma in the top layer. **(C)** The PRF clot. **(D)** The PRF membrane was ready to be placed into the defect

The PRF membrane was prepared according to the Choukroun protocol [22]. A 10 ml autogenous venous blood sample was drawn and taken into a sterile tube without the addition of any anticoagulant. The tubes were immediately placed in a centrifuge device.

for 10 min at 3000 revolutions per minute (rpm). The blood sample was separated into three layers at the end of the centrifugation process: the upper straw-colored acellular plasma layer; the middle layer included fibrin clot in which platelets were stuck; and the red lower layer included red blood cells (RBCs). The lower attached RBCS was separated and discarded by using surgical scissors. The middle fraction was collected and packed to form the PRF membrane. (Fig. 2)

**Fig. 3:**
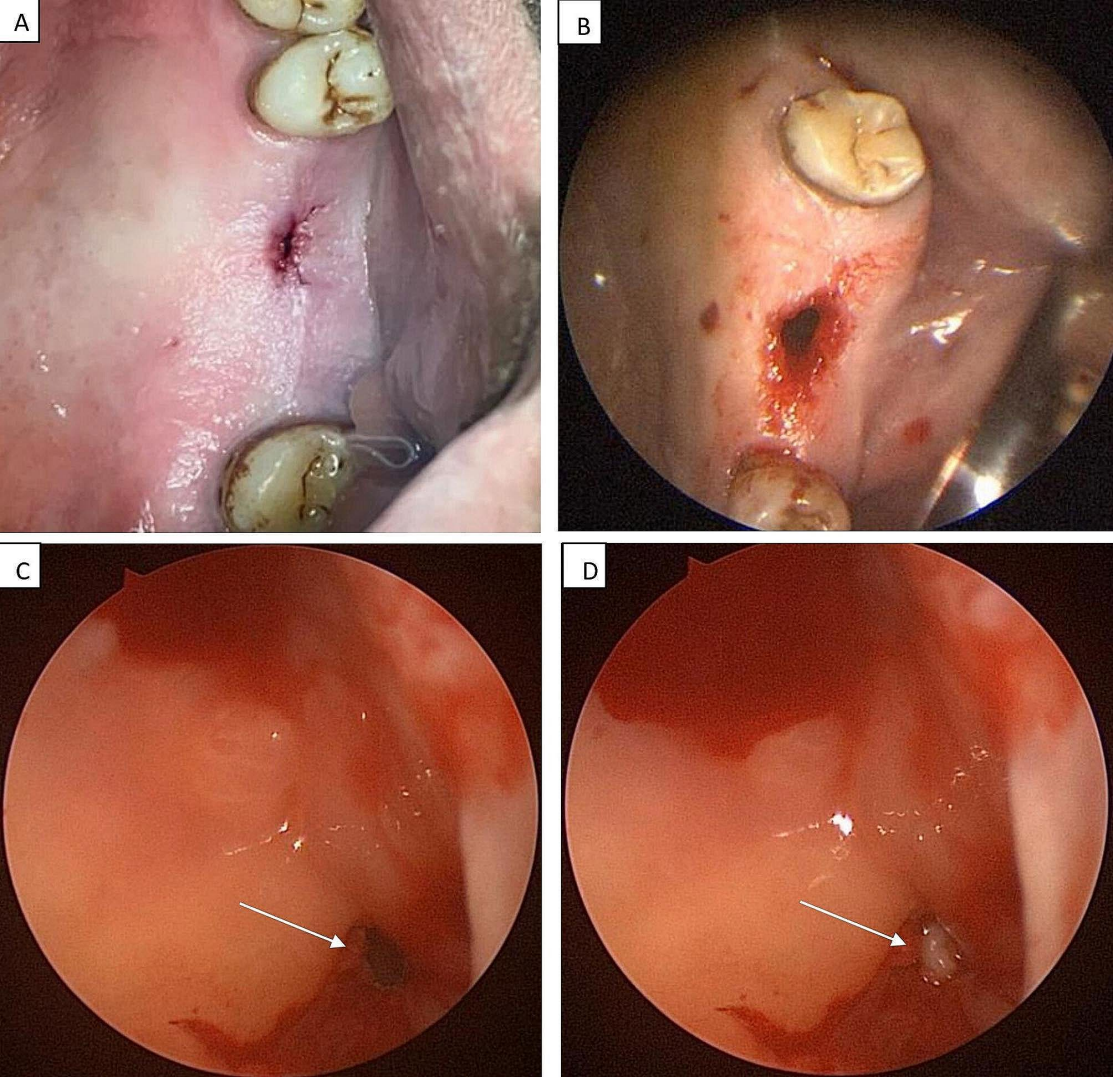
**(A) ** Preoperative clinical view showing chronic OAF related to the extracted maxillary 1st molar. **(B)** Intraoral approach; Fistulectomy with proper curettage. **(C)**. Endoscopic view of the defect during the operation. **(D)** Application of the PRF membrane to close the defect

**Fig. 4:**
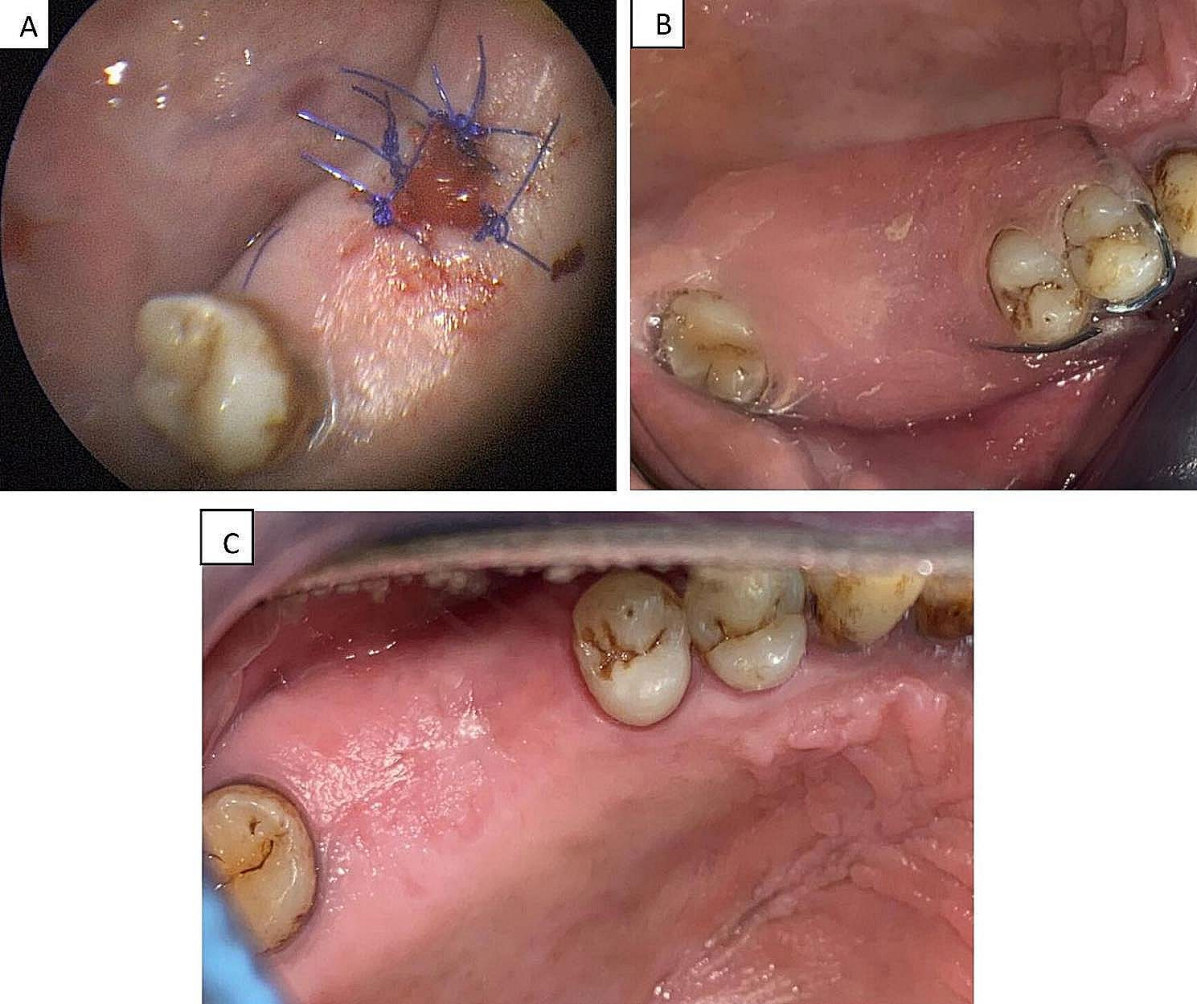
**(A)** The site of the OAF was filled with manipulated PRF membranes that were adapted over the defect, and secured with sutures. **(B)** The insertion of a prefabricated acrylic splint. **(C)** A clinical image showing complete closure of the OAF on day 14 during the follow-up phase

Then, the communication was closed by the application of a PRF membrane under the endoscopic control, which was introduced intraorally through the defect into the floor of the MS directly at the affected site of the Schneiderian membrane, and it was fixed by sutures with the surrounding oral mucosa to prevent any displacement. To ensure adequate closure, another two PRF membranes were placed into the defect between the palatal and buccal mucosa and secured with sutures by using a needle holder. Additionally, the edges of the oral mucosa were stitched. Finally, a well-fitted acrylic splint was inserted to cover the PRF membranes. (Figures 3 and 4)

### Postoperative and follow-up phase

Each patient was given certain instructions, including wearing the splint continuously until healing occurred (approximately 14 days(, nasal irrigation with saline for 5 days postoperatively, and eating a soft diet. Any activities that can increase intraoral or intranasal pressure such as smoking, drinking through straws, blowing their noses, and coughing while closing their mouths should be prevented. The following medications were prescribed for 5 days postoperatively: Augmentin 1gm twice daily, metronidazole 500 mg every 8 h (Flagyl: manufactured by GlaxoSmithKline, UK.), diclofenac potassium 50 mg every 8 h (Cataflam: manufactured by Novartis, Switzerland), a nasal decongestant as xylometazoline 0.1% two times per day (Otrivin: manufactured by GlaxoSmithKline, UK), and chlorohexidine 125 mg/100 ml antiseptic mouthwash 3 times per day (Hexitol: manufactured by Arabic Drug Company, ADCO.). It was recommended that all patients maintain good oral hygiene and regularly rinse their mouths.

### Primary outcome measurement

All patients had regular follow-up appointments until 24 weeks postoperatively. The following parameters were evaluated during that period:

The primary clinical outcomes; included the evaluation of soft tissue wound healing and complete closure of the fistula on the 1st, 7th, and 14th postoperative days using the clinical healing score (CHS) [23]. The sum of the five criteria represents a score called CHS, which ranges from 0 to 5, where 0 indicates excellent and complete epithelization of the wound, and vice versa. (Table 1) Additionally, if fluid regurgitation from the nose persisted or any complications occurred, it would be recorded. Postoperative pain was assessed on the 1st, 3rd, and 7th days postoperatively using a visual analogue scale (VAS) of 10 units [24]. 

(0 = the absence of pain; 1–2 = mild; 3–6 = moderate; 7–9 = extreme; and 10 = terrible).

**Table 1:** Clinical Healing Score (CHS).

Criteria	Score
Redness absent. Redness present.	0 1
Edema absent. Edema present.	0 1
Healthy granulation tissue was present. Healthy granulation tissue was absent.	0 1
A sign of epithelization was present. A sign of epithelization was absent.	0 1
Suppuration absent. Suppuration present.	0 1

### Secondary outcome measurement

Radiographic outcomes included examination of the sinus cavity, measurement of the size of the bone defect for each patient preoperatively with the aid of CT for comparison with the size of the defect 24 weeks after surgery, and calculation of the amount of bone formation [25]. 

## Statistical analysis

The data were collected and entered into a computer using the SPSS (Statistical Package for Social Science) program for statistical analysis (version 21) [26]. The data were collected as numerical or categorical, as applicable. The qualitative data are presented as percentages and numbers. The normality of the distribution was examined using the Kolmogorov-Smirnov test [27]. The quantitative data are presented as the minimum, maximum, mean, standard deviation, standard error of the mean, and median. During the sample size calculation, the beta error was accepted up to 20% with 80% study power. The alpha level was set to 5% with a significance level of 95%. Statistical significance was tested at a *p* value < 0.05.[28] The tests used were as follows:

- A repeated measures analysis of variance was used. Pairwise comparisons were performed with the Bonferroni correction.
- Comparisons were carried out between two studied dependent normally distributed subgroups using a paired-samples t test.

## Results

This study included nine patients who suffered from OAF and needed intervention. The age range reported in this study was 31 to 44 years, with a mean ± SD. of 38.22 ± 4.55 years. Based on the sex distribution, the present study revealed a male predilection as OAF occurred in 77.78% of the male patients and 22.22% of the female patients. There were no patients with diabetes mellitus. The OAF was due to the extraction of the upper 1st molar in 5 patients (55.56%), the upper 2nd molar in 3 patients (33.33%), and the upper 3rd molar in 1 patient (11.11%). The upper right quadrant was affected in 5 patients, while the left quadrant was affected in the other 4 patients. (Table 2)

**Table 2:** Demographic and clinical data of the study participants

Demographic data	n = 9
Age (years)	
Min-Max:	31.0–44.0 years
Mean ± SD.	38.22 ± 4.55 years
**Sex**:	
Male	7 (77.78%)
Females	2 (22.22%)
**Side affected**	
Right	5 (55.56%)
Left	4 (44.44%)
**location**	
First molar	5 (55.56%)
Second molar	3 (33.33%)
Third molar	1 (11.11%)
**Duration of OAF**	
Min-Max	4.0–24.0 months
Mean ± SD.	8.44 ± 6.27 months
**Systemic conditions& Habits**	
**Smoking**	
Yes	2 (22.22%)
No	7 (77.78%)
**Diabetes mellites**	
Present	0 (0.00%)
Absence	9 (100%)

SD: Standard deviation, Min-Max: Minimum – Maximum, n: Number of patients

All patients who were diagnosed with chronic odontogenic maxillary sinusitis were treated with the one-stage EMMA technique and OAF closure. In this study, 7 patients (77.78%) suffered from headache, and 4 patients (44.44%) had unilateral nasal obstruction preoperatively. Fluid regurgitation from the nose was observed in 3 patients (33.33%), and purulent discharge from the fistula was detected in 2 patients (22.22%). The duration of the OAF (months) in the patients ranged from 4.0 to 24.0 months, with a mean ± SD. of 8.44 ± 6.27 months. During the follow-up phase, symptoms such as headache, unilateral nasal obstruction, fluid regurgitation from the nose, and purulent discharge from the fistula completely disappeared postoperatively in all patients. (Table 3).

**Table 3:** Clinical symptoms of the patients

History	Preoperative		Postoperative	
**Fluid regurgitation from the nose** : - Absence - Present	n 6 3	% 66.67% 33.33%	n 9 0	% 100.00% 0.00%
**Headache:**				
- Absence	2	22.22%	9	100.00%
- Present	7	77.78%	0	0.00%
**Purulent discharge from the fistula:**:				
- Absence	7	77.78%	9	100%
- Present	2	22.22%	0	0.00%
**Unilateral Nasal obstruction** :				
- Absence	5	55.56%	9	100.00%
- Present	4	44.44%	0	0.00%

Pain was assessed using the visual analogue scale (VAS). On the 1st postoperative day, 3 patients (33.33%) had mild discomfort, while 6 patients (66.67%) had moderate pain. On the 3rd postoperative day, 8 patients (88.89%) had mild discomfort, and only one patient had moderate pain (11.11%). On the 7th postoperative day, 7 patients (77.78%) had no pain, and two patients (22.22%) had mild discomfort, which represented minimal pain. (Table 4) Pairwise comparisons of different time intervals revealed a statistically significant difference in the mean VAS score between the 7th postoperative day, and the 1st postoperative day (*p* < .001).

**Table 4:** VAS results for all patients to assess postoperative pain

Pain	1st day	3rd day	7th day	Test of sig.	*P*
Mean ± SD	3.78 ± 0.67	2.67 ± 0	0.22 ± 0.		
Min-Max	3–5	1–4	0–2		
Median	4	2	0	F_(df = 3)_ = 34.959*	*P <* .001*
**VAS (%)**:					
0: No pain	0.00%	0.00%	77.78%		
1–3:Mild discomfort	33.33%	88.89%	22.22%		
4–6:Moderate pain	66.67%	11.11%	0.00%		
**Sig. bet. Periods**	p_1_ = 0.126, p_2_ < 0.001*, p_3_ = 0.025*				

Pairwise comparison: adjustment by the Bonferroni correction for multiple comparisons

df: degree of freedom, Sig: significant, F: F for repeated-measures ANOVA test

p: *p* value for comparison between the different time intervals

p_1_: *p* value for comparison between the VAS score on the 1st and 3rd days

p_2_: *p* value for comparison between the VAS score on the 1st and 7th days

p_3_: *p* value for comparison between the VAS score on the 3rd and 7th days

*: statistically significant at *p* < .05

**Fig. 5:**
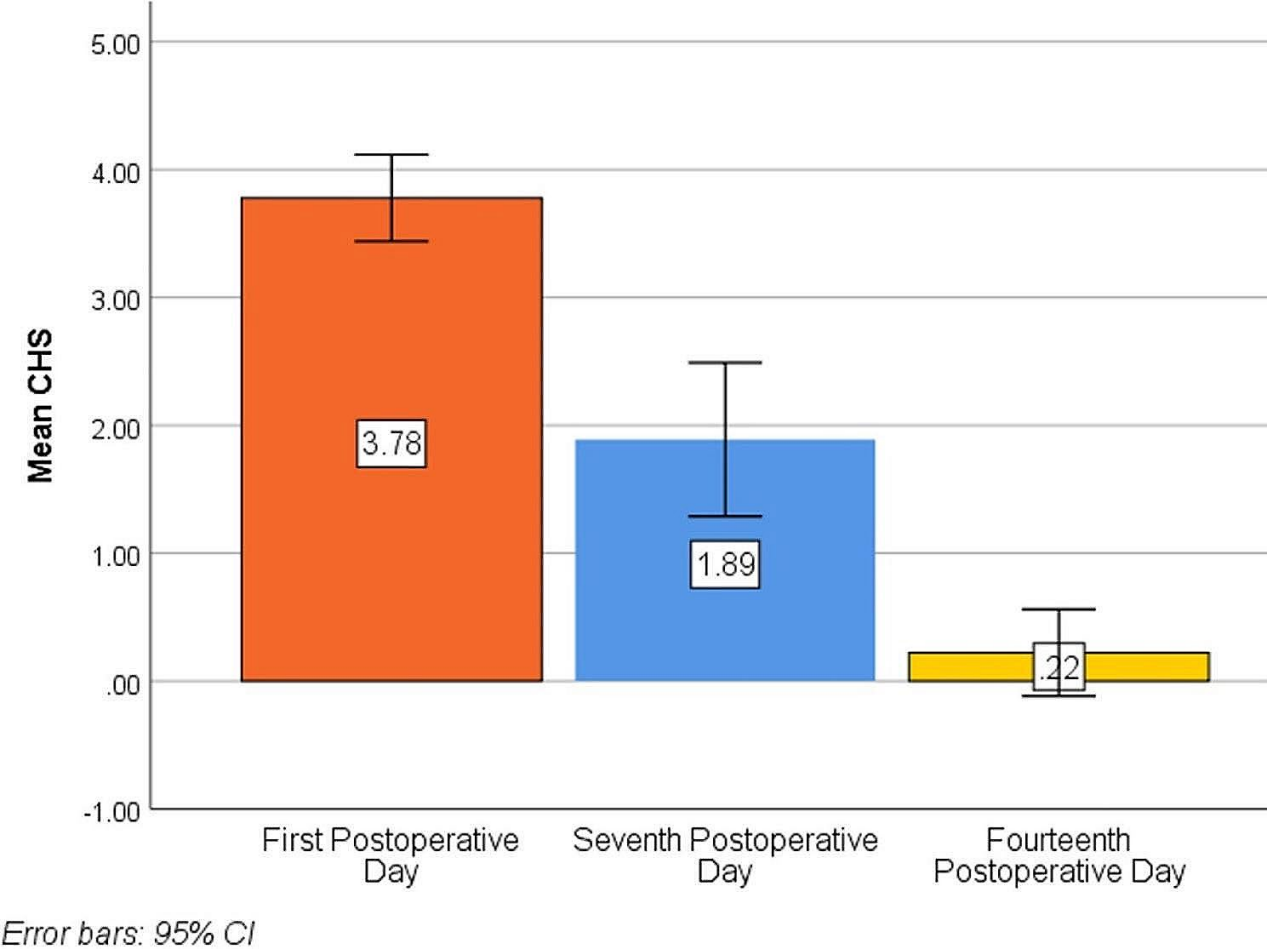
Simple bar chart of the mean CHS at different time intervals

Regarding soft tissue healing, on day 1 the CHS ranged from 3.0 to 4.0, while it ranged from 1.0 to 3.0 on day 7, and it ranged from 0.0 to 1.0 on day 14. Pairwise comparisons among different time intervals revealed a significant decrease in the CHS, which represented excellent healing (*p* < .001). (Table 5; Fig. 5) Additionally, the mean size of the bone defects ranged from 5.25 to 6.50 mm, with a mean ± SD. of 5.90 ± 0.38 mm preoperatively. After 24 weeks postoperative, the size of the bone defect decreased, and it measured between 0.00 and 1.25 mm, with a mean ± SD. of 0.58 ± 0.45 mm. Therefore, the amount of bone formation was calculated, and it ranged from 5.00 to 5.60 mm, with a mean ± SD. of 5.32 ± 0.20 mm. (Table 6)

**Table 5:** Evaluation of CHS on day 1, day 7, and day 14 after the operation

	Day 1	Day 7	Day 14	F	*P*
**Min-Max** **Mean ± SD**.	3.00–4.00 3.78 ± 0.44	1.00–3.00 1.89 ± 0.78	0.00–1.00 0.22 ± 0.44	F_(df=3)_ = 125.768	*<* 0.001*
**Sig. bet periods**	p _1_= 0.001*	p _2_<0.001*	p _3_<0.001*		

Pairwise comparison: adjustment by the Bonferroni correction for multiple comparisons

F: F for repeated-measures ANOVA test

p: *p* value for comparison between different time intervals

p_1_: *p* value for comparison between CHS at days 1 and 7

p_2_: *p* value for comparison between CHS at days 1 and 14

p_3_: *p* value for comparison between CHS at days 7 and 14

**Table 6:** The size of the bone defect preoperatively and 24 weeks postoperatively

	Pre. Size of defect (mm)	Post. size of defect (mm)	T	*P*	Amount of bone formation (mm)
**Min-Max** **Mean ± SD** **Median**	5.25–6.50 5.90 ± 0.38 6.00	0.00-1.25 0.58 ± 0.45 0.50	t = 78.532	*p*_*1*_ < 0.001.*	5.00-5.60 5.32 ± 0.20 5.30

Pre: preoperative, Post: postoperative, t: Paired sample t test

p: *p* value for comparison between the size of the bone defect preoperatively and postoperatively

*: statistically significant at *p* < .05

## Dissussion

Different studies have shown that the most common etiologic factor of OAF is complicated maxillary posterior tooth extraction, particularly extraction of the upper 1st molar, the upper 2nd molar, and finally the upper premolars [1, 2]. This might be due to the proximity of the maxillary roots to the thin MS floor in some cases. OAF was observed after extraction of the maxillary first molar in five patients, after extraction of the upper second molar in three patients, and after extraction of the wisdom molar in only one patient. The age range reported in this study was 31 to 44 years. Similarly, research conducted by Rasoul Gheisari et al. [29] has shown that OAF commonly occurs between the third and fourth decades of life and rarely occurs in children and adolescents. It has been suggested that the loss of teeth as individuals age could increase the possibility of OAF occurrence.

In this study, all patients suffered from unilateral odontogenic maxillary sinusitis related to the OAF. It was reported that 77.78% of patients suffered from headache and 55.56% of them had unilateral nasal obstruction. This finding agrees with a study by Georg Watzak et al. [3]. who reported unilateral maxillary sinusitis on the 4th day after sinus exposure in 60% of patients [3]. Considering the undesirable consequences of MS infection due to further penetration of bacteria and fungi into the sinus, closure of the OAF is a significant issue. A study performed by Soukaina Essaket et al. [30]. concluded that complete elimination of the fistulous tract, any pathology in MS, deteriorated polypoid mucosa, and any foreign body is mandatory for long term successful closure of the OAF and for decreasing the risk of failure [30]. 

Individuals who suffer from chronic sinus disease and who do not respond to conservative medical treatment need to undergo surgery, which includes the Caldwell-Luc procedure or the EMMA technique to manage sinusitis and provide adequate sinus drainage. EMMA enables the MS ostium to remain patent while meticulously preserving the mucosa, restoring normal function, and achieving efficient mucociliary clearance [7]. Recently, a one-stage approach was described to eliminate the infection completely, restore the physiological drainage of the sinus, and prevent the recurrence of maxillary sinusitis in patients who had OAF complicated by chronic sinus disease. In this study, all patients underwent a one-stage EMMA technique combined with the application of PRF membranes and were covered by an acrylic splint for more protection and prevention of contamination. With this technique, complete closure of the OAF with preservation of the vestibular sulcus depth, and successful sinus drainage were achieved in all patients without any complications. Additionally, widening of the natural MS ostium, and great resolution of sinusitis symptoms were observed with minimal postoperative pain, and edema that was verified through the results of clinical examinations and CT scans. A study performed by K. Joe Jacob et al. [6] revealed that EMMA was superior to Caldwell-Luc’s technique in regard to intraoperative and postoperative parameters such as patient comfort, pain, edema, epistaxis, bleeding, and hospital stay days for the treatment of chronic maxillary sinusitis [5, 6]. Usually, an antrostomy at the inferior meatus is created with the Caldwell-Luc procedure. A study performed by Yu Chen Huang et al. [31] and Mohamed S. Elmaradny et al. [32] suggested avoiding unphysiological inferior meatus antrostomy in diseased MS patients [31, 32]. 

Regarding the closure of the OAF, there are various techniques that can be used, but the choice between them is controversial. A study performed by Puria Parvini et al. [11] reported that using soft tissue flaps as buccal advancement flaps has several drawbacks, such as postoperative pain, reduction of the buccal sulcus, and edema due to mucoperiosteal flap reflection. In this study, PRF membranes and acrylic splint were used in all patients to minimize surgical trauma, and overcome complications such as flap necrosis, and the impossibility of repeating the surgical technique in case of clinical failure. Additionally, this technique preserves the height of the vestibular sulcus, which is a main concern in the planning of removable prosthodontic treatment. This finding is in accordance with that of G. Dell’Aversana et al. [12] who reported that the use of a PRF membrane alone provides excellent outcomes in the closure of oroantral communication with a low risk of complications, and promotes the development of mineralized tissue due to its osteoconductive properties [12, 33]. 

PRF has been used in various medical applications due to its simple preparation, as it does not require an anticoagulant [22]. It creates a dense three-dimensional fibrinous architecture matrix that can be sutured to the tissue [12]. A study by Ankit Sharma et al. [16] showed that PRF could stimulate osteogenesis and proliferation of osteoblasts, gingival fibroblasts, and periodontal ligament cells. These cell types are beneficial for bone formation and soft tissue regeneration [16, 18]. Similarly, a study by Zhanqi Wang et al. [34] showed that PRF promotes the continuous release of growth factors such as platelet- derived growth factor (PDGF), fibroblast growth factor, transforming growth factor β (TGF β-1), and vascular endothelial growth factor (VEGF), for up to 14 days. These factors promote angiogenesis and stimulate the healing process. Additionally, PRF increases vascularization, suppresses inflammation, and minimizes local pain [35, 36]. 

The outcomes of this study validate that the pain level was minimal, as 77.78% of the patients had no pain and 22.22% had mild discomfort on the 7th day postoperative. This finding agrees with the findings of Ahmed W [13]. and G. Dell’Aversana et al. [12] who reported that pain reduction may be due to the use of less invasive techniques and the anti-inflammatory activity of the PRF components. Additionally, research performed by Jonathan Meza et al. [37] on the effect of PRF in oral surgery has shown that PRF can minimize pain levels, and consequently decrease the need for analgesics [34, 37]. 

**Fig. 6:**
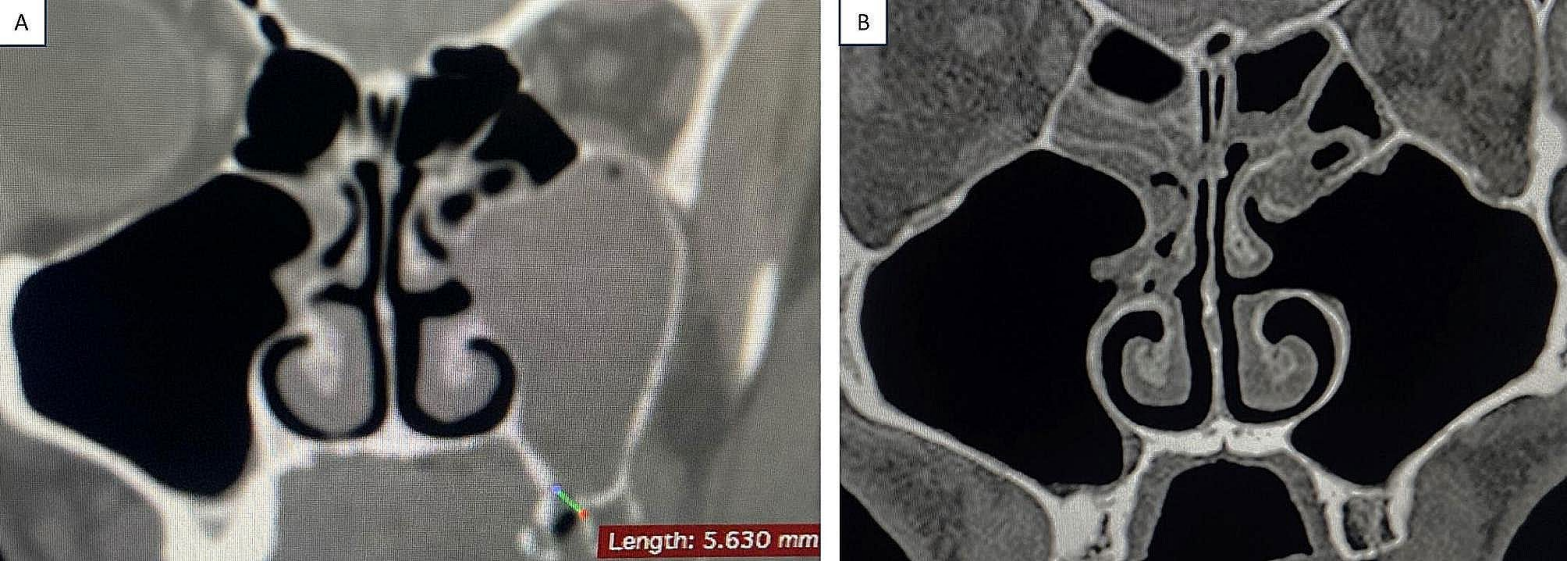
**(A)** Preoperative CT scan (coronal plane) showing an OAF, and discontinuity in the MS floor associated with complete opacification of the left MS. **(B)** Postoperative CT after 24 weeks revealed complete disappearance of the opacification at the left MS, and the fistula was obliterated

Radiographic follow-up was performed after 24 weeks postoperatively via CT scans. CT is considered the gold standard imaging technique and a reliable method for diagnosing MS pathology. It provides appropriate MS imaging due to its excellent resolution, accuracy, and ability to distinguish between soft tissue and bone [38]. A significant decrease in the size of the bone defect was found on postoperative CT compared with preoperative CT. Additionally, opacification in the ipsilateral MS cavity was observed on preoperative CT, but it disappeared completely in all patients on postoperative CT after EMMA which revealed a clear sinus with adequate drainage. (Table 6; Fig. 6) The limitation of this study is the small sample size, therefore more research is required to validate the findings. Within the limitations of this study, we achieved successful closure of the OAF, MS drainage, as all the excessive secretions were eliminated completely, and improvement in the quality of life of the patients when the EMMA technique combined with the application of PRF membranes were used.

## Conclusions

One-stage endoscopic middle meatal antrostomy (EMMA) with the application of platelet-rich fibrin (PRF) membranes and acrylic splint represent a reliable alternative technique for OAF closure and maxillary sinusitis relief that is associated with a lower incidence of complications and minimal postoperative pain.

## Data Availability

No datasets were generated or analysed during the current study.

## Footnotes

1 Manufactured by Karl Storz SE & Co. KG, Tuttlingen, Germany, https://www.karlstorz.com/.

## Abbreviations

OAF: Oroantral fistula
MS: Maxillary sinus
FESS: Functional endoscopic sinus surgery
EMMA: Endoscopic middle meatal antrostomy
PRF: Platelet rich fibrin
CHS: Clinical healing score
VAS: Visual analogue scale
